# The role of EUS in the diagnosis of early chronic pancreatitis

**DOI:** 10.1097/eus.0000000000000077

**Published:** 2024-08-21

**Authors:** Yaya Bai, Xianzheng Qin, Xiang Ao, Taojing Ran, Chunhua Zhou, Duowu Zou

**Affiliations:** Department of Gastroenterology, Ruijin Hospital, Shanghai Jiaotong University School of Medicine, Shanghai, China.

**Keywords:** EUS, Chronic pancreatitis, Early chronic pancreatitis, EUS-FNA; EUS-guided fine needle biopsy

## Abstract

The diagnosis of early chronic pancreatitis (ECP) is challenging due to the lack of standardized diagnostic criteria. EUS has been considered a sensitive diagnostic modality for chronic pancreatitis (CP), with advancements in technique such as EUS-guided fine needle aspiration and biopsy (EUS-FNA/FNB) being developed. However, their role in the diagnosis of ECP remains unelucidated. This review thereby aimed to provide an overview of the clinical landscape of EUS in the field of ECP.

## INTRODUCTION

Chronic pancreatitis (CP) is a fibroinflammatory syndrome characterized by recurrent inflammation, progressive fibrosis, and eventual irreversible tissue damage.^[1,2]^ Owing to the challenges with early detection and timely treatment, the disease burden of CP is high,^[3–5]^ with poor patient prognosis and quality of life.^[6,7]^

International consensus has recommended diagnosing CP based on morphology and has defined ECP as the stage with preserved pancreatic function and potentially reversible features.^[8]^ However, the clinical manifestations of ECP are unspecific, such as abdominal pain, and the test of pancreatic function is not always accessible in different medical centers. Therefore, the identification of ECP patients remains one of the most complicated issues for pancreatologists.^[9]^ Once ECP progresses to CP, damages become irreversible, and with the deterioration of both function and structure of the pancreas, more severe clinical outcomes would appear, such as pancreatic cancer, leading to the disability of patients. If ECP could be detected timely in clinical practice, those reversible impairments would be intervened earlier, which could change the management modality for patients with CP and improve the clinical outcome significantly through reversing the natural history of the disease. However, diagnosis by imaging alone is near impossible. Although genetic and environmental risk factors can contribute to the pathogenesis of ECP, their association with the etiology of the disease remains unelucidated.^[8]^ Therefore, an effective and standardized diagnostic approach for ECP remains in demand.

EUS has been considered the imaging modality of choice for CP due to its sensitivity toward morphological changes.^[10]^ In line with this, the revised Japanese Clinical Diagnostic Criteria for CP 2009^[11]^ has advocated for the use of EUS for the definitive diagnosis of ECP. The clinical utility of EUS has since been verified by several studies,^[12,13]^ and increased focus has been placed on novel techniques such as a multimodal EUS-based approach.^[14]^ Nevertheless, limitations remain in the lack of interobserver agreement with EUS for the diagnosis of CP.^[15–17]^ Furthermore, its correlation with surgical histopathology remains debatable. Trikudanathan et al. have assessed the correlation of standard EUS features for CP with surgical histopathology in a large cohort of patients with noncalcific CP (NCCP), suggesting that the correlation between standard EUS features and histopathology is poor in NCCP.^[18]^ EUS-guided fine needle aspiration (EUS-FNA) and biopsy (EUS-FNB) may facilitate with early detection of CP; however, their role in ECP has yet been established.

The aim of this paper is to provide a comprehensive overview of the clinical landscape of ECP and the current role of EUS in the field.

## EPIDEMIOLOGY AND DEFINITION OF ECP

The worldwide prevalence and incidence of ECP remain unclear. Based on a 2017 nationwide epidemiological study, ECP has been reported with a prevalence of 4.2 per 100,000 persons and an incidence of 1.0 per 100,000 persons in Japan. The male-to-female ratio was 1.32:1, with a mean age of 60.4 years, and a mean age at disease onset of 55.4 years. Idiopathic (47.7%) and alcoholic (45.0%) were the 2 most common etiologies. A considerably higher proportion of female and idiopathic cases were observed in ECP than in definite CP.^[19]^

Traditionally, accurate assessment of the earlier stages of CP was enhanced with the improvements of abdominal imaging in the 1980s. Afterward, the term “holding grade” was proposed to distinguish intermediate between acute and chronic pancreatitis, but this concept was eventually rejected and the term “probable CP” was used instead.^[20]^ Then, the term “probable alcoholic CP” was used by the 1996 Zürich Workshop^[21]^ to describe the early phases of CP. The revised Japanese Diagnostic Criteria for CP by the Japan Pancreas Society^[11]^ was the first to coin the terms “definite CP,” “probable CP,” and “early CP” for the classification of CP, by taking into account both clinical/functional and imaging features. The diagnostic criteria comprise of the following 6 items: (1) characteristic imaging findings, (2) characteristic histological findings, (3) repeated upper abdominal pain, (4) abnormal pancreatic enzyme levels in the serum or urine, (5) abnormal pancreatic exocrine function, and (6) continuous heavy drinking of alcohol equivalent to ≥80 g/d of pure ethanol. ECP is diagnosed based on the presence of ≥2 clinical signs from items (3)–(6), accompanied by positive findings on EUS or ERCP. Cases without supportive imaging findings are classified as “possible CP” and are indicated for repeat imaging within 3 months after the diagnosis [Table 1].

**Table 1:** The diagnostic criteria of CP and ECP.^[11]^

**Clinical diagnostic criteria for CP**
Diagnostic items for CP
(1) Characteristic imaging findings
(2) Characteristic histological findings
(3) Repeated upper abdominal pain
(4) Abnormal pancreatic enzyme levels in the serum or urine
(5) Abnormal pancreatic exocrine function
(6) Continuous heavy drinking of alcohol equivalent to 380 g/d of pure ethanol
Diagnostic criteria for ECP*
More than 2 items among (3)–(6) plus image findings of ECP
**Imaging findings of ECP**
Either a or b:
a. More than 2 features among the following seven features of EUS findings including at least one of (1)–(4)
(1) Lobularity with honeycombing
(2) Lobularity without honeycombing
(3) Hyperechoic foci without shadowing
(4) Stranding
(5) Cysts
(6) Dilated side branches
(7) Hyperechoic MPD margin
b. Irregular dilatation of >3 duct branches on ERCP findings

Patients with >2 items among (3)–(6) but without (1) and (2) are diagnosed as “possible CP” after ruling out other pancreatic diseases, with repeat imaging recommended within 3 months after diagnosis. Patients with imaging findings of early chronic pancreatitis plus either (3) or (4) in whom other pancreatic diseases are ruled out may have ECP; careful follow-up is required.

*The real nature of ECP will be clarified by long-term follow-up.

CP: chronic pancreatitis; ECP: early chronic pancreatitis; MPD: main pancreatic duct.

## HISTORY OF EUS IMAGING FOR THE DIAGNOSIS OF CP

The EUS features of CP have been comprehensively elucidated. The superiority of high-resolution EUS over conventional ultrasonography in visualizing the unique morphology of CP was first discovered in 1986 by Lees.^[22]^ This was further verified in same year by Sivak and Kaufman,^[23]^ who achieved a CP detection rate of 100% with EUS. Altogether, both studies emphasized the significance of parenchymal and ductal features for the workup for CP. Efforts have since been made to establish reliable EUS-based diagnostic criteria for CP, starting with the Minimum Standard Terminology in Gastrointestinal Endosonography proposed by the International Working Group in 1998.^[24]^ However, the reproducibility and applicability of this approach were subsequently challenged.^[25,26]^

The Rosemont criteria published in 2008 categorize EUS features as major and minor based on their significance toward establishing a diagnosis.^[27]^ The major EUS features were divided into major A and major B. The major A features include hyperechoic foci (>2 mm in length/width with shadowing) within the parenchyma and duct calculi (echogenic structure[s] within the main pancreatic duct with acoustic shadowing) of pancreas. Although the major B feature is lobularity (≥3 contiguous lobules = “honeycombing”) of the parenchyma. The minor features of the Rosemont criteria comprise hyperechoic foci (>2 mm in length/width, without shadowing), hyperechoic strands (≥3 mm in at least 2 different directions with respect to the imaged plane), lobularity (>5 mm, noncontiguous lobules), and (Pseudo) cyst (anechoic, round/elliptical with or without septations) of the parenchyma; or dilated duct (≥3.5 mm in body or >1.5 mm in tail), irregular duct contour (uneven or irregular outline and ectatic course), hyperechoic duct wall (echogenic, distinct structure >50% of entire main pancreatic duct in the body and tail), and dilated side branch (>3 tubular anechoic structures each measuring ≥1 mm in width, budding from the main pancreatic duct) of the duct. According to those endoscopic features, CP was divided into 4 types by the Rosemont criteria, including “consistent with CP” (1 major A feature + ≥3 minor features or 1 major A feature + major B feature or 2 major A features), “suggestive of CP” (1 major A feature + <3 minor features; or major B + ≥3 minor features; or ≥5 minor features, no major features), “indeterminate for CP”(major B alone or with <3 minor features; or 3 to 4 minor features, no major features), and normal (≤2 minor features, no major features). Thereafter, comparisons have been made between conventional and the Rosemont approach for the diagnosis of CP on EUS, which suggests that conventional criteria (CC) diagnose more cases of CP than Rosemont criteria (RC) when using 3-CC or when comparing 5-CC to “consistent with” CP by Rosemont, indicating that the Rosemont criteria are more stringent.^[28]^

A previous study has elucidated that one of the minor features of the Rosemont criteria, hyperechoic duct wall (echogenic, distinct structure >50% of entire main pancreatic duct in the body and tail) corresponding to the pathological features of ductal fibrosis, was one of the main EUS features of ECP.^[22]^ Nonetheless, more focus on the ability of EUS in the detection of mild changes in ECP is warranted.

## EUS FEATURES OF ECP

The early study by Lees^[22]^ identified progressive periductal fibrosis and recurrent lobular acute inflammation as the 2 main features of ECP. Wiersema and Wiersema^[29]^ subsequently proposed the relative homogeneity and hyperechogenicity of the pancreatic parenchyma in relation to the liver, as well as the irregularities in diameter of the pancreatic ducts in the head, body, and tail as EUS findings suggestive of ECP. It was further advised that age should be considered when defining normal pancreatic duct diameter, given that duct dilatation is an age-related process.^[30]^

Subsequently, Raimondo and Wallace^[26]^ gave an overview of the clinical utility of EUS in ECP, but the minimal changes in echotexture were difficult to interpret because there was no reliable gold standard confirmatory test; therefore, they emphasized the role of natural history to form a diagnosis of ECP. However, whether EUS is more sensitive to mild changes of CP or whether it perpetuates the overdiagnosis of ECP remains unclear.

It has been shown that progression from ECP, diagnosed based on minimal change features of CP on EUS (MCEUS), to definite CP is uncommon, and that such changes are often completely reversible.^[19]^ Many MCEUS features, including hyperechoic foci, hyperechoic strands, lobularity, and hyperechoic duct margins, are associated with fibrosis.^[31]^ Sheel et al.^[32]^ conducted a retrospective single-center study of the initial evidence for CP, with reassessment after follow-up (January 2003–November 2016), giving supportive longitudinal data about the diagnosis of ECP. In this research, the median (IQR) time to progression from MCEUS to either radiologically or histologically established CP was 30 (18.75 *vs.* 36.5) months. Of all patients with MCEUS features of ECP, only a third progressed to definite CP over a median of 3 years; this supports the view that MCEUS features may be a variant of normal rather than diagnostic of ECP. Therefore, they suggest that patients with MCEUS features are not stigmatized with a diagnosis of ECP especially if the EUS is performed within a year of an attack of acute pancreatitis and judicious clinical follow-up of at least 30 months is undertaken to monitor for progression to CP. In a word, MCEUS findings have limited sensitivity and specificity for the diagnosis of ECP.

## THE PATHOLOGICAL FEATURES OF ECP AND ITS CORRELATIONS WITH EUS CRITERIA

Theoretically, histology is considered gold standard for the diagnosis of CP. However, the sampling of affected tissue, particularly in the context of ECP, is often challenging. With increased recognition of EUS as a sensitive diagnostic modality for CP, exploration of its correlation with histopathology is warranted and has been done by several studies.

Walsh et al.^[33]^ in 1992 described ECP as minimal change CP and defined the condition as the presence of severe abdominal pain of pancreatic origin, but there were a few with minimal or equivocal findings on pancreatic investigation and in whom the etiology of their pancreatic disease was elusive. In this study, 16 patients underwent pancreatectomy after the failure of conventional treatment, and the pathological features were characterized by chronic inflammatory changes accompanied by subtle noninflammatory changes, including duct proliferation, duct complex formation, adenomatous nodules, and acinar cell atrophy, and cytoplasmic vacuolation. Finally, 9 patients were currently pain-free after resection or were very much improved.

The retrospective study by Albashir et al.^[34]^ involving 25 patients demonstrated that EUS score and endoscopic pancreatic function test (ePFT) results significantly correlated with the degree of histological fibrosis. The definitions of ECP were set as follows: EUS score, ≥4 of 9 standard criteria; ePFT peak bicarbonate concentration, <80 mM; and fibrosis score, ≥2. In addition, EUS showed a sensitivity and specificity of 84% and 100%, respectively, and significant improvement in sensitivity was achieved with the combined use of EUS and ePFT. Altogether, the study demonstrated that EUS and ePFT are comparable in terms of diagnostic performance for ECP.

In the study by Sekine et al.^[35]^ involving 12 patients who underwent pancreaticoduodenectomy for distal bile duct cancer without accompanying pancreatitis, 7 cases with abnormal EUS findings included in the diagnostic criteria for ECP were seen. In particular, the accuracy of lobularity seen on EUS was observed to be high (83.3%–91.7%) for pathological findings of the pancreatic parenchyma (inflammatory cell infiltration, atrophy of acinar cells, and fibrosis), whereas the accuracy of the hyperechoic margin of the pancreatic duct on EUS was high (83.3%) for pathological findings of the duct (wall thickness).

However, various conditions that associate with mild fibrosis, such as aging, diabetes, advanced renal disease, and elevated immunoglobin G4, may manifest similarly in terms of histology or morphology to ECP.^[8]^ The study by Trikudanathan et al.^[18]^ involving 68 patients with noncalcific CP demonstrated a significant but poor correlation between standard EUS features for CP and surgical histopathology (*r* = 0.24, *P* < 0.05). Monachese et al.^[36]^ hypothesized that natural history may be used as a gold standard to assess the predictive value of EUS and ePFT for the development of overt CP changes on computed tomography/magnetic resonance cholangiopancreatography. Their study found that EUS and ePFT carry the potential in predicting disease progression among patients presenting with abdominal pain of suspected pancreatic origin and with nondiagnostic cross-sectional imaging.

Further large-sample prospective studies remain warranted to clarify the correlation of EUS with histopathology in patients with suspected ECP.

## THE DIAGNOSTIC VALUE OF EUS-FNA/FNB IN ECP

EUS-FNA/FNB allows for the cytological and histological diagnosis of focal lesions, and has played a significant role in the differential diagnosis of pancreatic masses.^[37,38]^ Hollerbach et al.^[39]^ supported the role of EUS-FNA in ruling out CP. In the study by Albashir et al.,^[34]^ the 84% sensitivity and 100% specificity observed in comparison to histology for the detection of early fibrosis suggested that EUS with FNA may play a potential role in the diagnosis and staging of CP. The German S3-consensus guideline by Hoffmeister et al. ^[40]^ has recommended EUS-FNA for the workup of autoimmune pancreatitis. The 2020 ACG clinical guideline^[41]^ further suggested histological examination as the gold standard to diagnose CP in high-risk patients with strong clinical and functional evidence of CP but inconclusive imaging results.

However, the role of EUS-FNA/FNB in ECP, specifically, remains unelucidated. In guidelines such as those from Japan^[1,42]^ and European,^[10]^ emphasis has only been placed on EUS for the diagnosis of CP and ECP, with no mention of EUS-FNA/FNB. The ACG clinical guideline^[41]^ indicates histological examination in the case of inconclusive diagnoses, while simultaneously highlighting the limitations of this approach in terms of sampling error, biopsy-related complications, and the subjective nature of histologic interpretation. Similarly, the Consensus Guidelines for Chronic Pancreatitis by the International Working Group ^[8]^ suggested that the potential for EUS-FNA in the diagnosis and staging of CP, but emphasized the technical demands and operator-dependent accuracy associated with this approach [Table 2].

**Table 2:** Different guidelines on the application of EUS-FNA/FNB in CP or ECP.

No.	Guidelines	Country	Published Time	Statement
1	English language version of the S3-consensus guidelines on chronic pancreatitis: Definition, etiology, diagnostic examinations, and medical, endoscopic, and surgical management of chronic pancreatitis	International	2015	Cytological or histological fine EUS-FNA can be recommended to differentiate between autoimmune pancreatitis and other pancreatic diseases.
2	English language version of the S3-consensus guidelines on chronic pancreatitis: definition, etiology, diagnostic examinations, and medical, endoscopic, and surgical management of chronic pancreatitis	International	2015	EUS–guided fine needle biopsy (EUS-FNB) provides a cytological and/or histological diagnosis of focal lesions.
3	United European Gastroenterology evidence-based guidelines for the diagnosis and therapy of chronic pancreatitis (HaPanEU)	European	2017	EUS-FNB can be considered as the most reliable procedure for detecting malignancy.
4	Guidelines for the Diagnostic Cross Sectional Imaging and Severity Scoring of Chronic Pancreatitis	International	2018	It is possible that EUS with fine needle aspiration (FNA) can be supporting the diagnosis and staging of CP.
5	ACG Clinical Guideline: Chronic Pancreatitis	American	2020	We suggest histological examination as the gold standard to diagnose CP in high-risk patients when the clinical and functional evidence of CP is strong, but imaging modalities are inconclusive (conditional recommendation, very low quality of evidence).

EUS-FNB: EUS-guided fine needle biopsy; CP: chronic pancreatitis; ECP: early chronic pancreatitis.

Nonetheless, several studies have explored the role of EUS-FNA/FNB in nonfocal CP. DeWitt et al.^[43]^ evaluated the utility and the safety profile of transgastric EUS-guided Trucut biopsy in 45 patients with suspected CP (≥3 EUS criteria), and recommended against the approach due to potential complications, including acute pancreatitis and persistent abdominal pain without enzyme elevation, and limited diagnostic yield. The prospective study by Iglesias García et al. ^[44]^ on the efficacy of EUS-FNB for the histological diagnosis of ECP was terminated due to safety concerns and poor diagnostic yield, although a conclusion was made that EUS-FNB can be technically feasible for patients with indeterminate CP diagnosis on EUS [Table 3].

**Table 3:** Different studies on the clinical application of EUS-guided biopsy in ECP.

No.	Title	First Author	Published Time	Journal	Study Type	Inclusion Criteria	Number of Included Patients	Intervention	Conclusions
1	EUS-guided Trucut biopsy of suspected nonfocal chronic pancreatitis	DeWitt et al.	2005	Gastrointest Endosc	Prospective, single-center	Age, 18–60 y; ≥3 mo of unexplained mid-epigastric and/or periumbilical pain; previously known or suspected CP (regardless of severity) on radiographic evaluation	30	Transgastric EUS-TCB for suspected CP (≥3 EUS criteria) ERCP by an endoscopist blinded to the EUS results 1-wk post-EUS	Transgastric EUS-TCB of suspected nonfocal CP infrequently demonstrates histologic CP in clinically suspected disease. This technique is not currently recommended for evaluation of these patients due to associated complications and limited diagnostic yield.
2	EUS-guided fine needle biopsy (FNB) with the Procore™ needle provides inadequate material for the histological diagnosis of early chronic pancreatitis	Iglesias-García et al.	2018	Rev Esp Enferm Dig	Prospective, cross-sectional, single-center	Age >18 y; indeterminate EUS findings for the diagnosis of CP according to the Rosemont classification	The study was stopped after 11 patients were included due to safety concerns and poor diagnostic yield.	EUS-FNB of the body of the pancreas using Procore™ needles	EUS-FNB is technically feasible in patients with indeterminate EUS findings. However, the diagnostic yield is poor, and there is a nonnegligible risk of complications.

ECP: early chronic pancreatitis; CP: chronic pancreatitis: TCB: Trucut biopsy.

Altogether, the role of EUS-guided FNA/FNB in the diagnosis of ECP remains unclear. Further large-sample prospective studies are needed to explore this research topic.

## ADVANCEMENTS IN EUS TECHNIQUES FOR THE DIAGNOSIS OF ECP

Ongoing efforts have been placed in the development of diagnostic techniques to enable the accurate assessment of pancreatic fibrosis. EUS elastography, which provides a quantitative measure of tissue elasticity, has recently emerged as a potential diagnostic modality for ECP. In the study of Kuwahara et al.,^[45]^ they found that the “mean value” of EUS elastography (EUS-EG) gradually decreased from normal, indeterminate for CP, suggestive of CP to consistent with CP (based on Rosemont criteria). There was a significant negative correlation between the “mean value” and the number of EUS features. Meanwhile, it was suggested that hyperechoic foci with shadowing and lobularity with honeycombing maintained their independent diagnostic findings. In conclusion, EUS-EG provided objective information to support EUS features.

The contrast-enhanced harmonic EUS (CEH-EUS) technique offers information on vascularization and indicates blood flow patterns of normal and diseased tissue.^[46]^ It has been proposed to improve the differential diagnosis for CP^[10]^. In the study of Seicean et al.,^[47]^ 148 patients were evaluated to assess whether CEH-EUS-FNA is superior to standard EUS-FNA for specific diagnosis of solid pancreatic masses and what factors affect the diagnostic rate. Eventually, they found that EUS-FNA and CEH-EUS-FNA showed diagnostic sensitivities of 85.5% and 87.6%, respectively (not significantly different), and the combined sensitivity of the 2 passes was 93.8%. However, the role of CEH–EUS in the diagnosis of ECP requires further investigation.

The secretin endoscopic pancreatic function test (ePFT) has been investigated for its role in detecting ECP. Lara et al.^[48]^ demonstrated that sampling of pancreatic juice 20-minute after secretin administration showed a marginal sensitivity for CP. However, the authors highlighted that CP diagnosis should not rely on ePFT alone. Furthermore, the lack of correlation was observed between duodenal aspirate volume and peak bicarbonate concentration, which contrasts with current knowledge on secretin-stimulated magnetic resonance cholangiopancreatography (sMRCP), which is a noninvasive test of pancreas exocrine function based on pancreas duct compliance and duodenal filling. Further studies are thus warranted to explore the role of ePFT in this field.

Domínguez-Muñoz et al.^[14]^ employed a multimodal EUS-based approach involving EUS criteria of CP, elastography, ePFT, and main pancreatic duct compliance in a single test. Their proposed model not only enabled the morphological and functional evaluation of the pancreas, but also allowed for the detection of mild pancreatic abnormalities in patients with suspected ECP and inconclusive EUS results.

By far, artificial intelligence (AI) has been commonly used for differential diagnosis of CP, pancreatic cancer (PC), and autoimmune pancreatitis (AIP) in previous studies. Săftoiu et al.^[49]^ used neural network analysis to assess the accuracy of real-time EUS elastography in pancreatic lesions, and subgroup analysis suggested that differential diagnosis between PC and CP was still the main diagnostic problem. Recently, Marya et al.^[50]^ have reported that the convolutional neural network (CNN) could distinguish autoimmune pancreatitis (AIP) from CP with a sensitivity of 94% and specificity of 71%. Kuwahara et al.^[51]^ have investigated the efficacy of their AI model using EUS images of multiple types of pancreatic masses, revealing that the per-category sensitivities (95% CI) of CP were 0.78 (0.52–0.94) and 1.00 (0.61–1.00) in the test cohort and validation cohort, respectively, which means that AI model may distinguish pancreatic carcinomas from nonpancreatic carcinomas, but external validation is needed. However, the application of AI in the diagnosis of ECP needs to be classified. Future studies including high-quality randomized controlled trials (RCTs) and implementation of AI-assisted EUS in clinical practice are needed for early detection of pancreatic diseases,^[52]^ which could be a new direction for future scientific researches. Regardless, further prospective studies are needed to enhance the diagnostic accuracy of ECP.

## PROSPECT

Recently, new nonimaging approaches have been proposed. Adam et al.^[53]^ identified and independently validated a plasma- and serum-based metabolomic signature for the diagnosis of CP, which may be considered the benchmark for the development of a routine laboratory test for CP.

In the latest research of Lee et al,^[54]^ serum immune profiles of patients, including acute pancreatitis (AP), recurrent acute pancreatitis (RAP), CP, and chronic abdominal pain (CAP), were compared with healthy controls based on Olink immunoassay technology. Finally, they found that 33 immune markers were differentially expressed in the combined pancreatitis groups (AP, RAP, and CP) compared with controls. Meanwhile, compared with CAP, the Th17-related chemokines and cytokines, C-C motif chemokine 20 (CCL20), and interleukin 17A (IL17A) were upregulated in CP. Moreover, “macrophage classical activation signaling pathway” and proinflammatory upstream signaling (tumor necrosis factor [TNF] and interleukin 1β [IL-1β]) had gradually increasing heat intensity patterns with pancreatitis disease progression along the continuum of AP, RAP, and CP. This work provided new insights into the distinct immune markers that could serve as potential biomarkers to differentiate the varying pancreatitis disease states, probably including ECP.

A blood-based microRNA panel developed by Xin et al.^[55]^ demonstrated that when applied on clinical serum samples, hsa-miR-320a-d was accurate in predicting late CP, whereas hsa-miR-221 and hsa-miR-130a were accurate in predicting ECP with areas under the curve (AUCs) of 100.0% and 87.5%, which suggested that the panel has the potential to be applied clinically for early diagnosis of CP.

In conclusion, the exploration for new specified diagnostic biomarkers for ECP is in demand and more prospective researches investigating into ECP is necessary.
